# Contextual influences on the impact of a peer worker-led self-stigma program for people with mental health issues: protocol for an interventional implementation science study

**DOI:** 10.1186/s43058-020-00002-y

**Published:** 2020-02-25

**Authors:** Michelle Banfield, Alyssa R. Morse, Amelia Gulliver

**Affiliations:** Centre for Mental Health Research, Research School of Population Health, The Australian National University, Acton, ACT 2601 Australia

**Keywords:** Mental health, Peer work, Self-stigma, Recovery, Honest Open Proud, Consolidated Framework for Implementation Research, Implementation

## Abstract

**Background:**

Despite significant recent reforms, Australia’s mental health system faces substantial service and workforce shortages, and progress on the reorientation of services to a recovery focus is also slow. Implementing recovery-focused programs led by mental health peer workers is one way of addressing these issues, but uptake of these programs in Australia is patchy and not well evaluated.

This project will investigate the implementation of a peer-led mental health self-stigma program across three diverse settings in an Australian capital city. The project aims to (1) examine the processes and contextual influences on successful implementation of peer work roles and (2) evaluate the impact a peer worker-led program has on individuals and services.

**Methods:**

The project will use an interventional implementation science approach: small-scale, researcher-led implementation of the Honest, Open, Proud program to explore contextual influences, and implementation, service and individual outcomes. The Consolidated Framework for Implementation Research (CFIR) will be used to guide investigation of contextual factors. Implementation outcomes (feasibility, fidelity, acceptability, sustainability) and service outcomes (safety, person-centeredness and effectiveness) will be examined in interviews with a range of staff within the services, checklists of adherence to program protocols and analysis of administrative data. Individual quantitative outcomes will include self-stigma, recovery and quality of life, measured at baseline, end of program and follow-up. Qualitative enquiry will focus on experiences with the peer worker and program.

Quantitative analyses will comprise change scores on service and individual outcome measures. Where possible, differences between settings and the effects of potential confounds will be tested using multi-level linear models, which will account for clustering of individuals within programs and settings. Qualitative data will be analysed using a framework approach, which is an effective way of combining inductive and deductive coding to test and refine a thematic frame.

**Discussion:**

Recovery-focused, peer-led programs have the potential to provide a unique contribution to the management of mental health issues. Currently, there is little information to guide successful implementation of these roles. This project will provide important preliminary data on the factors that affect implementation and the impact peer workers can have.

**Trial registration:**

ANZCTR - Trial Registration ID: ACTRN12619001312134. Registered 26/09/2019

Contributions to the literature

- Research indicates that peer work can have a positive impact on outcomes and satisfaction with care for both the consumers and the peer workers.
- The potential for augmenting the workforce with peer workers has strong potential to improve current services’ focus on recovery and alleviate substantial burden on the mental health system.
- The current protocol outlines a project that will provide critical evidence using implementation science methods that can ensure the optimal delivery of peer work in the Australian context.

## Background

The Australian mental health system is undergoing a significant period of reform and change. Government reforms are shifting service delivery from a one-size-fits-all approach to a stepped-care model, and the recent roll-out of the National Disability Insurance Scheme (NDIS) has changed the funding and availability of many support programs. Increasing the capacity of mental health peer workers has been identified as a key strategy to support the mental health workforce and to improve continuity of care in this time of change [1–4], making the development and promotion of the mental health peer workforce a priority for the Australian Government. In 2014, the National Mental Health Commission National Review of Mental Health Programmes and Services recommended establishing National Mental Health Peer Workforce Development Guidelines to define the roles and functions of peer workers and provide guiding principles for their training, employment and support [4]. However, it is important that policymakers and service planners include rigorous evaluation in their implementation of these programs to develop our understanding of how peer work produces beneficial outcomes.

Peer work is a rapidly growing area that can have a positive impact on both consumer outcomes and satisfaction with care, as well as for the peer workers themselves [5–7]. International evidence shows that peer work is able to produce meaningful change for mental health consumers [5, 7], but implementation of peer work programs is piecemeal, and the link between programs with research evidence and current practice is poor [8]. To ensure the optimal delivery of peer work in the broader Australian mental health system, it is vital to trial the implementation of international evidence-based peer recovery programs.

Peer work is a rapidly growing industry internationally [5, 6, 9, 10]. Peer support occurs informally in support groups or partnerships, but it has also developed formally into voluntary and paid positions within consumer-operated and standard healthcare services: peer work [9]. Peer workers can perform a variety of conventional roles within services, including coaching, advocacy, case management, outreach, crisis support and delivering assertive community treatment and social support programs [11]. They use their lived experience of mental illness to inform their practices in providing emotional, social and instrumental support to other consumers [7, 12], although a recent systematic review found equivocal evidence for the benefits of peers over other types of workers in these positions [11].

However, peer workers can also be employed in unique, peer-specific roles, in which they use their own lived experience to promote recovery through mutuality, reciprocity and role-modelling [6]. In these circumstances, recent reviews conclude that peer support workers can produce a range of benefits for consumers, including an increased sense of independence and empowerment, improved self-esteem and confidence, improved social support and community integration, breaking down perceived stigma and fostering a sense of hope through positive role-modelling [5, 7]. Peer workers have also reported experiencing personal benefits from their occupation, including improved self-esteem and a sense of empowerment [6, 7].

One way in which peer work has progressed in recent years has been through the development of manual-based peer-led programs that specifically target peer worker outcomes such as stigma, empowerment and recovery. Honest, Open, Proud is one such program, developed by renowned mental health consumer and principal investigator on the National Consortium for Stigma and Empowerment, Professor Patrick Corrigan. Originally called Coming Out Proud due to its strong basis in the gay and lesbian movement, the program focuses on identity and disclosure as a means for reducing internalised stigma and promoting empowerment [13]. Consisting of three “lessons” and a booster session, Honest, Open, Proud peer facilitators guide participants through a workbook and activities that explore whether they identify as someone with a mental illness (or other similar terms), the pros and cons of disclosure and how to construct their story in a way that is safe for everyone if they do choose to disclose, particularly if they choose to be completely open such as through public speaking [13, 14].

Early randomised controlled trial evidence from diverse populations, including adolescents [15] and adults with schizophrenia [16] indicates that Honest, Open, Proud is effective at reducing disclosure-related stress and self-stigma, and improving recovery attitudes, but effects on other outcomes such as empowerment are less clear [15–17]. As the program becomes popular internationally, including Australia, it is important to add to the evidence of its effectiveness and explore how it performs for different groups and in different settings. In addition, the role of the program as a catalyst for authentic peer work is also important to establish.

### Aims

Thus, the current study has two primary aims:

1. Examine the processes and contextual influences on successful implementation of peer work roles; and
2. Evaluate the impact the peer worker-led recovery program, Honest, Open, Proud has on individuals and on services.

This study protocol addresses the Standard Protocol Items: Recommendations for Interventional Trials (SPIRIT) checklist [18] and the Standards for Reporting Implementation Studies (StaRI) checklist [19]. Additional file 1 presents the SPIRIT Checklist, and Additional file 2 presents the StaRI checklist.

## Method

### Study design

The project will use an interventional implementation science approach: small-scale, researcher-led implementation of the Honest, Open, Proud program to explore outcomes and contextual influences [20]. Consistent with Proctor et al.’s conceptual model for implementation research [21], the project will explore intervention strategies, implementation strategies and outcomes for implementation, services and individuals.

Better Together is being conducted according to the principles of community-based participatory research, which are particularly appropriate in implementation research that focuses on the relevance and uptake of interventions. The investigators all identify as mental health consumers and work in peer-identified roles and the research questions were developed by consumers and carers [1]. The intervention was developed and tested by a mental health consumer researcher, and local training for facilitators will be provided by an experienced social worker and peer supervisor, who was involved in program development and is the program’s Australian champion.

Research protocols and materials were co-developed with stakeholders via the ACACIA Consumer and Carer Advisory Group to ensure the nature and style of questions is appropriate for participants. In particular, consistent with consumer and carer preferences [22], qualitative feedback opportunities will be semi-structured to balance specific questions with the opportunity for participants to relate their experiences in their own words.

A pilot study focused on testing methods and measures for the individual level outcomes and preliminary investigation of selected implementation outcomes will be conducted with university students prior to commencement of the main study.

### Setting

Data collection is planned for three distinct settings within one Australian capital city: community-based public mental health services, a university and a recovery-focused adult mental health learning organisation (Recovery College).

Peer workers have recently been introduced into the city’s public mental health system, with positions in a variety of hospital and community-based settings. Group-based programs are already undertaken by at least one peer worker and opportunities exist to extend implementation to other peer workers. The program will be delivered and evaluated during usual peer work sessions.

The second planned setting is a university. The investigator team, who are all peer researchers and educators, will facilitate the Honest, Open, Proud program for staff and students of the university as part of the wellbeing program. The program will be held on campus at times agreed with participants to accommodate study and work responsibilities.

The third planned setting is a mental health Recovery College. The investigators and/or peer educators within the Recovery College will facilitate the program as part of 2019 second semester course offerings. The Recovery College has a specific focus on co-production and the importance of peer-led recovery, making it an ideal comparator for the other settings, in which these concepts are being introduced retrospectively.

### Program

Honest, Open, Proud consists of nine tasks in three lessons, plus a follow-up booster session (see Table 1) [23]. The delivery format is flexible, but is usually delivered in three weekly 2-h sessions, with the 2-h booster approximately 3–4 weeks later. The pilot will be delivered in this format, and participants queried about other delivery formats such as an intensive 1-day workshop or 1-hour weekly sessions delivered across a semester in the post-program feedback. Final choice of delivery format will be guided by participant preference when groups are formed.

**Table 1:** Honest, Open, Proud lessons and tasks

Lesson	Tasks
Lesson 1: Considering the pros and cons of disclosing	Task 1 - Do you identify yourself as a person with a mental illness
	Task 2 - Consider the pros and cons of disclosure
Lesson 2: Different ways to disclose	Task 1 - Different ways to disclose
	Task 2 - To whom might you disclose
	Task 3 - How might others respond to your disclosure
Lesson 3: Telling your story	Task 1 - How to tell your story
	Task 2 - How did it go?
	Task 3 - Honest, Open, Proud through peer support
	Task 4 - Putting it all together
Booster	Follow-up 1 - The decision to disclose
	Follow-up 2 - Peer support programs
	Follow-up 3 - What has changed

The full program manual and workbook are available at www.comingoutproudprogram.org.

Each session comprises information about stigma and disclosure, worksheets and activities that encourage participants to think about their own views and choices, to weigh up pros and cons and to make informed choices. The group format allows for discussion of key points and practice sessions for disclosure and story-telling in a supportive environment, facilitated by peers with experience being “out”.

### Participants

Study participants comprise three main groups at each setting: peer workers, other staff and people with experience of mental illness.

It is anticipated that approximately eight peer workers will participate, including the three study investigators. All peer workers will be invited to contribute data to the study in the form of reflective notes on their experiences and/or participation in an interview.

Up to five staff members in each setting involved in the peer worker program directly or indirectly will be invited to participate by direct invitation from one of the investigators. During implementation of the program, investigators will develop a list of leadership, clinical and administrative staff at each location who may be able to provide insight into the key implementation issues. Participants will be purposively sampled from these lists to provide a range of views on the five implementation construct domains of the Consolidated Framework for Implementation Research (CFIR) [24].

The third key group is people with lived experience of mental illness. As an exploratory study, no specific sample size calculations have been undertaken for individual outcomes. Based on medium-sized effects observed in previous studies trialling the Honest, Open, Proud program [17], we will aim for a minimum of three groups comprising eight participants per study setting (*n* = 72). Data collected during pilot testing of the intervention will also be included (total *N* = 80).

There are no specific exclusion criteria. However, the program and associated research project measures are all in English and approach the issues from a Western conceptualization of stigma and mental illness. As such, this may prove challenging for people from different cultural or language backgrounds. We will not specifically exclude these people from participating, but they may require extra assistance to take part.

### Outcomes and measures

#### Primary outcome

The primary outcome is the success of the implementation of peer work roles across the settings. This will be investigated using the CFIR [24], which provides a “menu of constructs” associated with successful implementation and allows systematic assessment of barriers and facilitators and the generation of theory. The CFIR consists of five core domains:

- Intervention characteristics
- Outer setting
- Inner setting
- Characteristics of individuals
- Process

Using the tools provided at www.cfirguide.org, including the interview guide builder, specific constructs of focus to investigate contextual influences for the current study across the five domains will be selected in collaboration with the ACACIA Consumer and Carer Advisory Group. Expected constructs include adaptability and complexity of the intervention, external policy and incentives, inner setting culture and implementation climate, individual stage of change and all process constructs.

Measures of specific implementation outcomes (feasibility and acceptability) to be completed after program delivery are provided in Additional file 3. The evaluation questions are designed to gather information from program participants on the Honest, Open, Proud program, the delivery format (number and length of sessions), and the peer facilitators. Fidelity will be measured using the program’s fidelity scale (available at http://www.comingoutproudprogram.org/images/Honest_Open_Proud_Fidelity_2.9.2017-min.pdf), scored by one investigator at each session.

Integral to the participatory nature of both the program and the project is information about the peer delivery. The project investigators will record notes and reflections on their own experiences delivering the program as part of data collection. Other peer workers involved in program delivery will be invited to do the same and to participate in an interview at the completion of each Honest, Open, Proud program to generate rich, cumulative data on the implementation process.

#### Secondary outcomes

At the service level, outcomes of interest are safety, person-centeredness and effectiveness. These will be investigated via interviews with staff and analysis of administrative data, including quality and safety monitoring, and routine outcome measurement statistics such as service level consumer experience data [e.g. the Your Experience of Service (YES) questionnaire [25]] to assess overall changes to these figures following introduction of the peer workers and program.

Table 2 presents the secondary outcomes at the individual level. These outcomes will be self-stigma, measured by both a specific self-stigma scale [Internalized Stigma of Mental Illness Inventory [26]] and a disclosure scale [Coming Out with Mental Illness Scale [27]], empowerment [Empowerment Scale [28]], recovery [Self-Identified Stages of Recovery [29]], and quality of life [Personal Wellbeing Index [30]]. A brief distress screener [Distress Questionnaire 5 [31]] and personal and clinical characteristics are also included.

**Table 2:** Individual level secondary outcome measures

Construct	Measure	No. of Items	Example items	Rating scale
Self-stigma	Internalized Stigma of Mental Illness Inventory [26]	10	For each question, please mark whether you strongly disagree (1), disagree (2), agree (3), or strongly agree (4): “I can have a good, fulfilling life, despite my mental illness”	4-point scale Range: 1 (strongly disagree) to 4 (strongly agree).
Self-stigma	Coming Out with Mental Illness Scale [27]	21	Are you out about your mental illness? (yes = complete page 2, no = complete page 3). Page 2: I came out of the closet to gain acceptance from others. Page 3: In the future I will come out of the closet to gain acceptance from others.	Page 2, 3: 7-point scale Range: 1 (strongly disagree) to 7 (strongly agree).
Disclosure	Individual disclosure items (author-created)	4	Have you disclosed your mental illness to your family and friends? “In general, how distressed or worried are you in terms of secrecy or disclosure of your mental illness to others?”	7-point scale Range: 1 (not at all) to 7 (very much).
Empowerment	Empowerment Scale [28]	28	“I see myself as a capable person” “I can pretty much determine what will happen in my life”	4-point scale Range: 1 (strongly disagree) to 4 (strongly agree).
Recovery	Self-Identified Stages of Recovery [29]	5	Part A: Of the five statements above, which one would you say most closely describes how you have been feeling over the past month about life with the illness? Part B: “I am confident that I will find ways to attain my goals in life”	Part A: 1 (I do not think people can recover from mental illness. I feel that my life is out of my control, and there is nothing I can do to help myself) to 5 (I feel I am in control of my health and my life now. I am doing very well and the future looks bright) Part B: 6-point scale Range: 1 (strongly disagree) to 6 (strongly agree).
Quality of life	Personal Wellbeing Index [30].	8	The following questions ask how satisfied you feel: “How satisfied are you with what you are achieving in life?”	10-point scale Range: 1 (no satisfaction at all) to 10 (completely satisfied).
Psychological distress	Distress Questionnaire 5 [31]	5	In the last 30 days: “My worries overwhelmed me” “I had trouble staying focused on tasks”	5-point scale Never (1) to always (5)
Evaluation questions	Author-created items	12	What did you like about the program?	Open-ended question

### Data collection

**Fig. 1:**
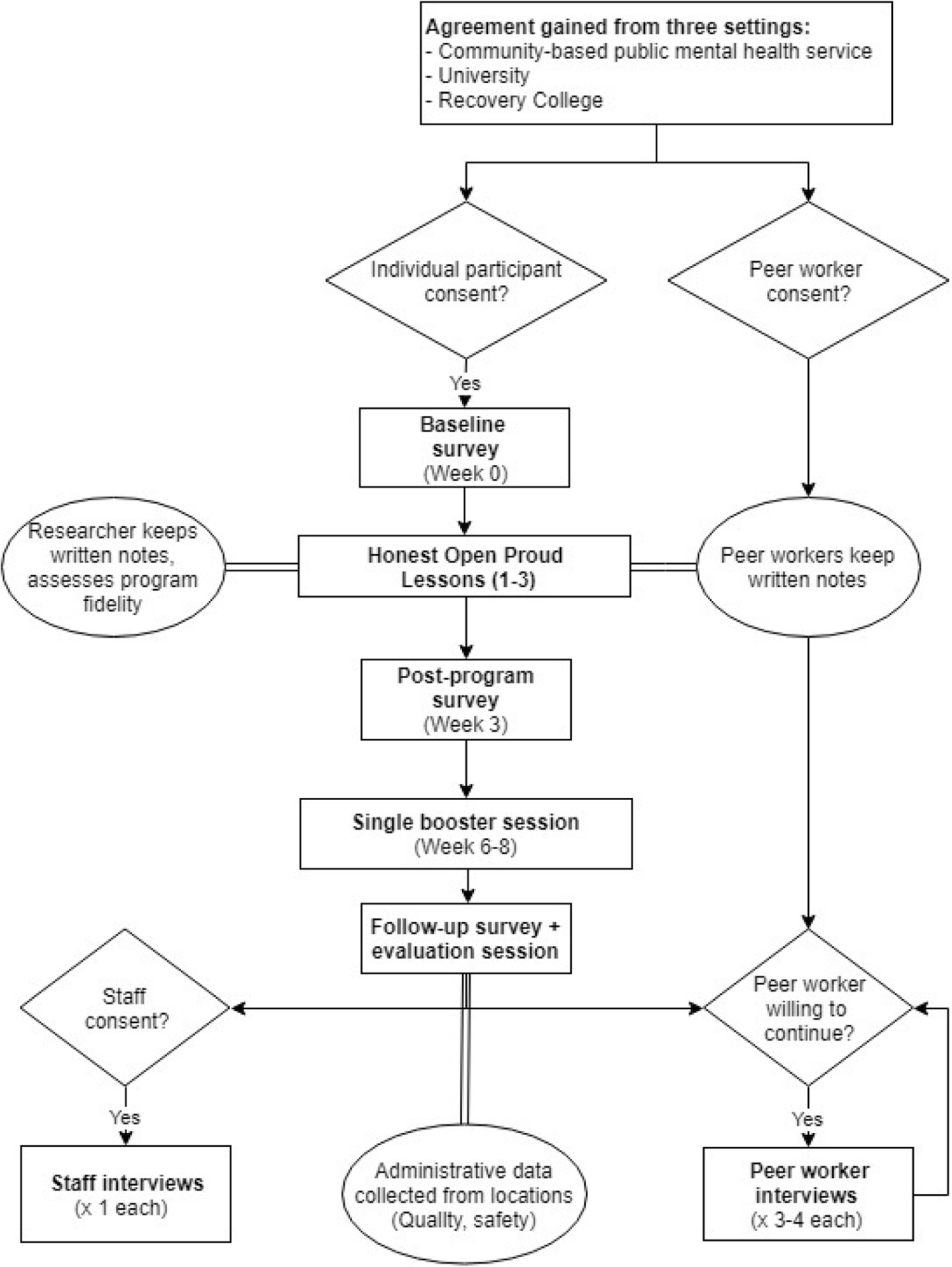
Study flow

Figure 1 presents the study flow.

Table 3 presents the assessments and their delivery time points in the study.

**Table 3:** Assessment time points

	Construct	Baseline	Post-program (3 weeks)	Follow-up (after booster session ~ 8 weeks)
Primary outcomes				
	Implementation success across settings			✓
Secondary outcomes				
Service level	Safety			✓
	Person-centredness			✓
	Effectiveness			✓
Individual level	Demographic data	✓		
	Self-stigma	✓	✓	✓
	Disclosure	✓	✓	✓
	Empowerment	✓	✓	✓
	Recovery	✓	✓	✓
	Quality of life	✓	✓	✓
	Psychological distress	✓	✓	✓
	Program evaluation (peer workers)			✓
	Program evaluation (staff)			✓
	Program evaluation (HOP participants)			✓

#### Program participants

Baseline measures including demographic and limited clinical data will be completed upon recruitment to the study and return of the consent form, prior to commencing the Honest, Open, Proud program. Questionnaires will be available both on tablets managed by the research team (using Qualtrics) and in paper form, according to participant preference. To avoid error introduced by participant management of codes, questionnaires will be identified by participant first name (or pseudonym) and last initial. Participants will be advised about the use of names during the initial contact about the program so that they may elect not to participate if uncomfortable with identified data collection. Names will be removed and replaced by a code after matching of questionnaires is complete. Outcome measures will be completed at the conclusion of Lesson 3 (3 weeks) and again at the conclusion of the booster session (6–8 weeks after baseline).

A brief feedback session (half-to-one hour) will be conducted at the end of the booster sessions to gather additional information on participant experience. People who did not participate in the outcome measurement may elect to participate in the feedback session by completing written informed consent at this point of the study. Participants may take part in a face-to-face discussion or elect to answer the same open-ended questions in a brief, online survey.

Total participation time, including program sessions, outcome measures and the feedback session is approximately 10–12 h across 8 weeks. Data collection is anticipated to commence in October 2019 and be completed early in 2020.

#### Peer workers

Data for peer workers facilitating the program will comprise reflective notes on experiences of delivering the program and 30-min interviews conducted at the conclusion of each Honest, Open, Proud course (after the booster session). Peer workers will be asked to keep written or typed notes on their experiences to be submitted for inclusion in the research data at the conclusion of data collection. Interviews will follow a semi-structured protocol, guided by the CFIR constructs, and will be digitally recorded for professional transcription. Willingness to continue participation will be checked with peer workers at each participation occasion. Participation may extend for 6 months from October 2019 and comprise three to four interviews in that period. Facilitation of the program will take approximately 8 h plus preparation time. Estimated research data collection time is approximately 2 h of notes per course (total of 6 h) plus up to 2 h of interview time (30 min per interview).

#### Staff

Data for staff in the settings in which the program will be delivered will comprise one interview of approximately 60 min. Interviews with up to five staff members at each site will follow a semi-structured protocol, guided by the CFIR constructs, and will be digitally recorded for professional transcription. Participation for staff members will be on a single occasion only. Data collection from staff is anticipated to commence late in 2019, after at least one Honest, Open, Proud course has been completed at the site.

#### Administrative data

During study setup, a request for aggregated administrative data on quality, safety and consumer experiences will be made to the data governance bodies of each location. To minimise issues of confidentiality and delays due to complex permissions, these data may be restricted to publicly available reports on performance. The purpose of examining administrative data is to supplement primary data from staff observations of service-level changes and individual participant outcomes.

### Analysis

Quantitative data will comprise program scores of fidelity, overall service scores on measures of safety, effectiveness, patient-centeredness and experience, and individual measures of self-stigma, empowerment, recovery and quality of life. Due to the exploratory nature of the study, it is expected that quantitative data will be primarily descriptive and comprise change scores across time. Where possible, differences between settings and the effects of potential confounds will be tested using multi-level linear models, which will account for clustering of individuals within programs and settings.

All qualitative data will be analysed using a framework approach [32]. Framework analysis, a comprehensive and systematic qualitative analysis method driven by the observations of participants, is particularly appropriate for studies with applied research questions that are concerned with people’s experiences of a phenomenon and the influence of contextual factors. Data will be interrogated for the CFIR constructs [24] (contextual factors) and implementation, service and individual outcomes specified in the conceptual model [21]. The framework will be elaborated with inductive coding of concepts not well-described by the high level dimensions of the model.

## Discussion

The current protocol describes a study that uses an implementation science approach to evaluate a small-scale implementation of a program in three distinct settings in an Australian context. The primary aim of the study is to evaluate the success of the implementation of the program, with secondary aims to examine other related implementation factors including feasibility, fidelity, acceptability and sustainability of the program, service-level outcomes of safety, person-centeredness and effectiveness of the program, and individual-level outcomes for the effectiveness of the program on aspects such as self-stigma, recovery and quality of life. Peer work can have a positive impact on both consumer outcomes and satisfaction with care, as well as for the peer workers themselves [5–7]. Further, augmenting the workforce with this important and accessible group has strong potential to alleviate substantial burden on the mental health system [3, 4] and improve services’ recovery focus [5–7]. This project will provide critical implementation evidence to ensure the optimal delivery of peer work in the broader Australian mental health system to achieve these benefits.

## Supplementary information

**Additional file 1.** SPIRIT 2013 Checklist: Recommended items to address in a clinical trial protocol and related documents.

**Additional file 2.** Standards for Reporting Implementation Studies: the StaRI checklist for completion.

**Additional file 3.** Evaluation questions (for discussion or written response).

## Data Availability

Not applicable.

## Abbreviations

ACACIA: The ACT Consumer and Carer Mental Health Research Unit
ACT: Australian Capital Territory
ANZCTR: Australian New Zealand Clinical Trials Registry
CFIR: Consolidated Framework for Implementation Research
NDIS: National Disability Insurance Scheme
YES: Your Experience of Service
