# Supplementary material for: Contextual influences on the impact of a peer worker-led self-stigma program for people with mental health issues: protocol for an interventional implementation science study
Source: Implement Sci Commun. 2020 Feb 25;1:26. doi: 10.1186/s43058-020-00002-y (PMC7427925; doi:10.1186/s43058-020-00002-y)
Supplement: Supplementary file 3 — Additional file 3. Evaluation questions (for discussion or written response). [file 43058_2020_2_MOESM3_ESM.docx]

# Evaluation questions (for discussion or written response)

## Program acceptability & feasibility

Did you enjoy the “Honest, Open, Proud” program?

What did you like about the program?

What didn’t you like about the program? Why?

Is there anything you would change about the program?

How would you like it to change?

How would you rate the number of sessions: [too few/just right/too many]

Why?

How would you rate the amount of content in each session: [too little/just right/too much]

Why?

How would you rate the length of sessions? [too short/just right/too long]

Why?

Do you think the program is a good fit for [setting]?

Why/why not?

Did you connect with or relate to the program facilitators?

Why/why not?
